# Psychological and Demographic Predictors of Vaping and Vaping Susceptibility in Young Adults

**DOI:** 10.3389/fpsyg.2021.659206

**Published:** 2021-08-17

**Authors:** Grace E. Teah, Tamlin S. Conner

**Affiliations:** Department of Psychology, University of Otago, Dunedin, New Zealand

**Keywords:** e-cigarette, electronic cigarette, vaping, survey, personality, mental health, young adult, socioeconomic status

## Abstract

**Background:**

The use of electronic nicotine delivery systems (ENDS), also known as vaping, is becoming popular among young adults. Few studies have explored the psychological factors that predict ENDS use and susceptibility in young adults, in addition to known demographic predictors.

**Method:**

In a cross-sectional survey design, 521 young adults (37% male), ages 18–25 from the United States, were recruited via Amazon’s Mechanical Turk (MTurk) in 2019, to answer an online survey measuring demographic characteristics and psychological characteristics related to mental health and the Big Five personality traits. The survey also included measures of ENDS ever-use, current use, and susceptibility (never users open to trying ENDS), which we predicted from the demographic and psychological measures using independent and multiple binary logistic regression analyses.

**Results:**

Of those surveyed (*n* = 521), 282 (54.1%) were ENDS ever-users, 93 (17.9%) were current ENDS users, and 61 (11.7%) were ENDS susceptible; 62 (11.9%) were current smokers. Demographically, young adults lower in adulthood socioeconomic-status (SES), not pursuing education further than high school, and current smokers were more likely to be ENDS users. Psychologically, young adults higher in anxiety and lower in conscientiousness more likely to have ever-used ENDS. Lower conscientiousness further predicted current ENDS use and ENDS susceptibility.

**Conclusion:**

In this sample of MTurk workers, young adults with experience in vaping were more demographically and psychologically vulnerable than young adults with no experience in vaping. Young adults interested in vaping, but without prior experience, were less conscientious than their non-interested peers. Interventions to target vaping use should focus on economically disadvantaged young adults and those lower in conscientiousness.

## Introduction

The use of electronic nicotine delivery systems (ENDS), known colloquially as ‘vaping’ or e-cigarette use, continues to grow in popularity, particularly among young adults aged 18–25 (McMillen et al., 2014; Mirbolouk et al., 2018). Two studies have shown that 40% of undergraduates reported some lifetime ENDS use (usually defined as at least a single puff) (Lanza and Teeter, 2018). With growing concerns over increases in nicotine dependency for smokers who use ENDS, and for non-smokers developing nicotine addictions, it is timely to explore the factors associated with young adults’ ENDS use.

To date, much of the literature has focused on the demographic factors that predict ENDS use amongst young adults (Littlefield et al., 2015; Saddleson et al., 2015; Mirbolouk et al., 2018) whereas the psychological factors predicting ENDS use, such as mental health status and personality traits, are less clear. In this paper, we investigated the psychological and demographic factors in the same sample, to better understand the predictors of young adults’ ENDS use. We also investigated predictors of ENDS use susceptibility, which is the “the absence of a firm decision to not to use cigarettes” (e-cigarettes/ENDS), based on previous smoking susceptibility research (Pierce et al., 1996, p. 355).

Research has identified several demographic predictors of ENDS use in young adults including gender, ethnicity, university attendance, and socioeconomic-status (SES). Young adult males have been found to have higher intentions to use ENDS (Lee et al., 2017), and are more likely to use ENDS than young adult females (Choi and Forster, 2013; Littlefield et al., 2015; Saddleson et al., 2015; Temple et al., 2017; Mirbolouk et al., 2018). Moreover, individuals identifying with non-binary genders also have high levels of ENDS use (Mirbolouk et al., 2018), which reflects smoking statistics (ONS, 2020). There is mixed evidence linking ethnicity to ENDS use, with some research showing higher use in Hispanic and White populations (Temple et al., 2017), higher use in Hispanic and other racial minority university students (Sutfin et al., 2013), or no ethnicity differences (Littlefield et al., 2015). Also, ENDS use is quite common among university-attending young adults (Littlefield et al., 2015; Saddleson et al., 2015; Lanza and Teeter, 2018), but the association between university attendance and ENDS use is unknown as these studies only sampled student populations.

A further demographic predictor of ENDS use is SES. Individuals with lower SES have higher smoking rates (Barnett et al., 2009; Hiscock et al., 2012), and smoking is known to predict ENDS use in young adults (Saddleson et al., 2015). However, with ENDS use, prior research has found that adults with higher SES, or higher incomes are more likely to be aware of and use ENDS (Adkison et al., 2013; Glover et al., 2018). Given the high start-up costs of ENDS devices, yet a lower ongoing cost in comparison to cigarettes (Cheng et al., 2021), and the fact that health behaviors may diffuse from higher to lower SES individuals over time (Pampel, 2005), it is important to determine the role of SES in ENDS uptake and use. It is particularly important to determine if SES plays a different role in ENDS use than in smoking. Prior research has also not distinguished between childhood SES and adulthood SES in predicting ENDS use. Research has associated smoking in young adulthood with lower childhood family SES, however, this association could be explained by covariates (Patrick et al., 2012). If adulthood SES were a stronger predictor than childhood SES of ENDS use and susceptibility, this could suggest that current socioeconomic environments are more important than formative socioeconomic environments.

Research on the psychological predictors of ENDS use is beginning to provide valuable insights into young adults’ ENDS use. One observation is that young adults have different motivations for ENDS use than the general adult population, where young adults might be more motivated by curiosity and experience-seeking/sensation-seeking (Hampson et al., 2015; Kong et al., 2015; Trumbo and Kim, 2015), as well as taste and flavor (Pokhrel et al., 2015; Temple et al., 2017). By contrast, the general adult population tends to report using ENDS in an attempt to quit smoking (Etter and Bullen, 2011; Sussan et al., 2017; Rhoades et al., 2019). This difference means that psychological traits related to curiosity, interest, and openness to experience may be valuable to explore in relation to young adults’ ENDS use and susceptibility.

To date, personality characteristics, such as The Big Five Factors (openness to experience, conscientiousness, extraversion, agreeableness and neuroticism) (Goldberg, 1999), have not been extensively explored in relation to ENDS, despite known associations between personality and smoking. Three meta-analyses suggest that smokers are higher in extraversion (Munafò et al., 2007) and neuroticism (Malouff et al., 2006; Munafò et al., 2007), and lower in conscientiousness (Bogg and Roberts, 2004; Malouff et al., 2006) and agreeableness (Malouff et al., 2006) than non-smokers. Further research links higher neuroticism and lower conscientiousness to nicotine dependence among both African American and European-American men and women (Choi and Forster, 2014). This raises the question as to whether these same personality traits may predispose ENDS use. There has been one exploratory study on the personality predictors of ENDS use in 380 young adults, which showed no associations (Hittner et al., 2020). However, that study measured personality using the 20-item Mini-IPIP, which may not provide sufficient coverage of each personality trait and their underlying aspects.

Mental health factors such as stress, anxiety, and depression also require exploration in relation to ENDS use. Smokers are more likely to be mentally distressed, and report higher levels of perceived stress than non- or former-smokers (Ng and Jeffery, 2003). Similarly, there is a greater chance of anxiety, stress, depression, and substance use in ENDS-using young adults (Conway et al., 2018; King et al., 2018), as well as higher levels of perceived stress linked to ENDS and heated tobacco product use in young adults (Lee et al., 2019). While early research indicates that mental health factors relate to ENDS use comparably to smoking, further research is needed to understand associations between mental health factors and ENDS susceptibility.

Therefore, our study used a cross-sectional correlational design to explore both demographic and psychological factors in relation to young adults’ ENDS use and susceptibility. We administered a survey that measured ENDS ever-use, current use, and susceptibility along with a range of demographic factors (gender, ethnicity, university status, childhood and adulthood SES, current smoking), and psychological factors related to mental health (perceived stress, anxiety, depressive symptoms) and personality (curiosity/exploration and the Big Five personality traits). We utilized a full-scale personality inventory that measured both traits and aspects to provide more specificity to explore personality predictors of ENDS outcomes.

## Materials and Methods

### Ethics

The “Lifestyles of Young Adults” study was approved by the University of Otago Department of Psychology (Category B Ethics #D17/158), with oversight by the University of Otago Ethics Committee. Participants were provided with information regarding the study, and asked to provide informed consent before the survey began.

### Design and Participants

We recruited 800 young adults aged 18–25, via Amazon’s Mechanical Turk (MTurk) internet recruitment tool, between May and July 2019 to complete a broad survey on the “Lifestyle of Young Adults.” Respondents were required to be ages 18–25, living in the United States, have a Human Intelligence Task (HIT) approval rate > 90% to exclude bots, and to not have taken the survey previously run in 2017 and 2018. Of the 800 who consented and began the survey, 235 did not pass the survey attention checks, 22 stopped responding, seven provided incomplete data, four were found to have a response bias (answering all questions with the same response), and the first 11 participants were excluded because the ENDS survey questions were formatted incorrectly, leaving a sample of 521 participants. The survey took approximately 30 min and participants were remunerated with USD $1.50, which was the MTurk rate when the study was run in 2019. To reflect evolving employment situations, we have since increased our remuneration for subsequent MTurk research.

### Survey Measures

#### Demographic and Lifestyle Measures

The demographic information surveyed included age, gender, ethnicity, student and employment status, education level, and childhood and adult socioeconomic status. Lifestyle questions included one question measuring current smoking: ‘How often do you smoke cigarettes (rolled or filtered)?’ and participants responded either “I don’t smoke now’; ‘’Less than once a month’; ‘At least once a month’ ‘At least once a week’; ‘At least once a day.’ Participants who responded ‘At least once a month’ or more, were categorized as current smokers, all other participants were categorized as not current smokers. Supplementary Appendix 1 shows the demographic and lifestyle questions and response options used in the survey.

#### Psychological Measures

This study utilized five validated mental health and personality measures. Mental health included perceived stress using the Perceived Stress Scale (PSS) (Cohen et al., 1983), anxiety using the Anxiety Subscale of The Hospital Anxiety and Depression Scale (HADS) (Zigmond and Snaith, 1983), and depressive symptoms using the Centre for Epidemiological Studies Depression Scale (CES-D) (Radloff, 1977). Personality focused on trait curiosity, measured using the Curiosity and Exploration Scale-2 (Kashdan et al., 2009), and the Big Five traits and aspects measured using the 100-item Big Five Aspects Scale (BFAS) (DeYoung et al., 2007), which yields five trait scores (neuroticism, agreeableness, conscientiousness, extraversion and openness) and 10 aspect scores (two aspects per trait). Supplementary Table 1 provides detail on the psychological measures including response options, descriptive statistics, and alpha reliabilities.

#### Electronic Nicotine Delivery Systems Measures

Electronic nicotine delivery systems use was measured using two questions from the New Zealand International Tobacco Control Survey Wave 2 Questionnaire (ITC, 2018). ENDS ever-use was measured with one question: “Have you ever used an e-cigarette or vaping device, even one time?”, and participants responded either ‘Yes’ or ‘No.’ Current ENDS use was measured with one question: “How often do you currently use e-cigarettes or vaping devices?,” with the response options: ‘Not at all’; ‘Less than monthly’; ‘At least once a month, but not every week’; ‘At least once a week, but not every day’; ‘Every day.’ We did not display pictures of ENDS devices to participants or specify device types. For analysis, we dichotomized the current ENDS use variable by treating those who responded, ‘Not at all’ and ‘Less than monthly’ as ‘Not a current user’ and participants with all other responses categorized as ‘Current users.’

Electronic nicotine delivery systems susceptibility was measured using two questions modified from a validated smoking susceptibility index (Pierce et al., 1996). This measure has also shown predictive validity in ENDS use in adolescents (Cole et al., 2019; Seo et al., 2020). Those who responded ‘No’ to the ENDS ever-use question, were asked: “If a friend offered you his or her vape, would you puff on it?” and “Do you think you are likely to try a vape within the next 6 months?”, with these response options for both questions: ‘Definitely yes’; ‘Probably yes’; ‘Probably no’; ‘Definitely no.’ We classified people based on their responses in two ways. First, following (Pierce et al., 1996, p. 355), we created a two-group classification of ‘Not susceptible’ participants who responded ‘Definitely no’ to both questions and ‘Susceptible’ participants who endorsed other responses. This served as our primary measure of susceptibility. Second, following (Strong et al., 2015), we also created a three-group classification of ‘Not susceptible’ participants who responded ‘Definitely no’ to both questions, ‘Highly susceptible’ participants who responded ‘Probably yes’ or ‘Definitely yes’ to at least one question and ‘Moderately susceptible’ participants who did not endorse ‘Probably yes’ or ‘Definitely yes’ for either question and did not endorse ‘Definitely no’ to both questions.

### Data Preparation and Analyses

Data were prepared and analyzed using SPSS (v.26) (IBM Corp., 2019). Three binary primary outcome variables were created from the ENDS variables: ENDS ever-use (0 never use, 1 ever-use), ENDS current use (0 not used in past 30 days, 1 used in past 30 days or more), and ENDS susceptibility (0 not susceptible, 1 susceptible). All 521 participants were included in the analyses of ever-use and current use; a sub-sample of 239 participants (ENDS never users) were included in the analyses of susceptibility. We did not conduct analyses of dual-use in this paper, given the low number of dual-users (*n* = 29). We then conducted independent logistic regressions predicting the three binary primary outcomes from each of the demographic and psychological factors analyzed separately and multiple logistic regression models entering the demographic and psychological factors together to control for each other. Supplementary logistic regression models were also run predicting the secondary ENDS susceptibility measure based on the three group categorization (0 not susceptible versus 1 highly susceptible; 0 not susceptible versus moderately susceptible). Results present the odds ratios, confidence intervals, and significance values. For interpretation, we adjusted the *p* value downward from *p* < 0.05 to *p* < 0.005 to limit the number of false positives following Benjamin et al. (2018). A traditional correction such as Bonferroni was not appropriate due to correlated outcome measures. Supplementary data visualizations consisting of box plots and bubble plots were also created in R (v.3.6.3; R Core Team, Vienna, Austria, 2020) to show the dispersion of demographic and psychological measures for all participants, different ENDS users (never users, susceptible, current users), and current smokers.

## Results

### Descriptive Statistics

Table 1 presents participants’ demographic characteristics. Their mean age was 23 (SD 1.67), with 60% female. One-third identified as a racial minority or mixed ethnicity. Participants came from a range of backgrounds, with varying SES; there was a mix of students (44%) and non-students (56%), with the majority engaging in tertiary education beyond the high school level (79%). The number of current smokers was 62 (11.9%), compared to 8% of 18–24 years old American population who reported current smoking in 2019 (Cornelius et al., 2020).

**TABLE 1:** Descriptive statistics for the participant characteristics (*n* = 521) and the electronic nicotine delivery system (ENDS) measures.

Continuous measures	Mean (*SD*)	Range
Age	23.19 (1.67)	18.00–25.00
SES adulthood	3.63 (1.67)	1.00–7.00
SES childhood	3.73 (1.62)	1.00–7.00

**Categorical measures**	**Category**	***n* (% of) sample**

Gender	Female	313 (60.1%)
	Male	193 (37.0%)
	Gender diverse	15 (2.9%)
Ethnicity (Top 6)	White	342 (65.6%)
	Mixed	66 (12.7%)
	Black	38 (7.3%)
	Asian	30 (5.8%)
	Hispanic	29 (5.6%)
	Other	16 (3.1%)
Student/employment status	Student	229 (44.0%)
	Employed	234 (44.9%)
	Unemployed	58 (11.1%)
Highest attainment^1^	Not in further education, unemployed	36 (6.9%)
	Not in further education, employed	76 (14.6%)
	In further education	229 (44.0%)
	Completed further education, unemployed	22 (4.2%)
	Completed further education, employed	158 (30.3%)
Location	Urban	202 (38.8%)
	Suburban	237 (45.5%)
	Rural	82 (15.7%)
Smoking frequency	Don’t smoke now	442 (84.8%)
	Less than once a month	17 (3.3%)
	At least once a month	14 (2.7)
	At least once a week	12 (2.3%)
	At least once a day	36 (6.9%)
Current smoker^2^	Current smoker	62 (11.9%)
	Not a current smoker	459 (88.1%)

**ENDS survey measures**	**Category**	***n* (% of sample)**

Ever-use	Yes	282 (54.1%)
	No	239 (45.9%)
Vaping frequency	Not at all	377 (72.4%)
	Less than monthly	51 (9.8%)
	At least once a month	18 (3.5%)
	At least once a week	18 (3.5%)
	Everyday	57 (10.9%)
		***n* (% of Never Users)**
Vape susceptibility (Friend)	Definitely Yes	5 (2.1%)
	Probably Yes	16 (6.7%)
	Probably No	35 (14.6%)
	Definitely No	183 (76.6%)
Vape susceptibility (6-months)	Definitely Yes	1 (0.4%)
	Probably Yes	9 (3.8%)
	Probably No	33 (13.8%)
	Definitely No	196 (82.0%)

**ENDS analyzed measures**		

Ever-use	Yes	282 (54.1%)
	No	239 (45.9%)
Current use^3^	Current user	93 (17.9%)
	Not a current user	428 (82.1%)
Susceptibility (*n* = 239)^4^		
Based on two groups^5^	Susceptible	61 (25.5%)
	Not susceptible	178 (74.5%)
Based on three groups^6^	Highly susceptible	22 (9.2%)
	Moderately susceptible	39 (21.9%)
	Not susceptible	178 (74.5%)

*^1^Highest attainment was computed from combining information from the Student/Employment Status measure with the Level of Education measure (not shown). Further education was defined as any tertiary-level program (university, college, polytechnic) beyond the high school level.*

*^2^Current smoker defined as using at least once a month to once a day.*

*^3^Current use defined as past 30-day use (Vaping frequency at least once a month to everyday).*

*^4^Vape susceptibility measured only in the 239 participants who responded No to Ever-using a vape.*

*^5^Two groups defined as Susceptible (answered Definitely Yes, Probably Yes, or Probably No to at least one of the Friend and 6-months susceptibility questions) or Not Susceptible (answered Definitely No to both the Friend and 6-months susceptibility questions), based on Pierce et al. (1996).*

*^6^Three groups defined as Highly Susceptible (answered Definitely Yes, or Probably Yes to either the Friend or 6-months susceptibility questions), Moderately Susceptible (did not answer Definitely No to both the Friend or 6-months susceptibility questions, and did not answer Definitely Yes or Probably Yes to either the Friend or 6-months susceptibility questions) or Not Susceptible (answered Definitely No to both the Friend and 6-months susceptibility questions). These labels are equivalent to Highly Susceptible, Susceptible, and Committed Never User from Strong et al. (2015), respectively.*

Supplementary Tables 1, 2 present the descriptive statistics for the psychological variables and their correlation matrix. The mean score for perceived stress was 19.72 (SD 8.73), which indicates participants were moderately stressed on average. The mean score for anxiety was not especially high at 7.71 (SD 5.04) (where a score of 11 or higher is classed as an abnormal case), however, the mean score for depressive symptoms was 20.35 (SD 13.62), which is four points above the cut-off of 16 that indicates possible depression. In total, 56% of young adults in our sample scored a 16 or higher on the CES-D, indicating clinically significant symptoms. The personality measures were all within norms for the young adult population. The variables were correlated with each other in expected ways (Supplementary Table 2).

Table 1 also presents the ENDS measures. Of our sample, 282 (54.1%) had ever used an ENDS, and 93 (17.9%) were current ENDS users, which was greater than the number of current smokers (11.9%). Only 29 participants reported using both cigarettes and ENDS (5.6% dual users). There were 239 (45.9%) participants who had never used an ENDS, and of these, 61 were categorized as susceptible to ENDS (reflecting 11.7% of the full sample, or 25.5% of the never users subsample). Of these 61 susceptible participants, 22 were highly susceptible and 39 were moderately susceptible to ENDS.

### Independent Logistic Regressions

Table 2 presents the independent logistic regressions for the demographic predictors. Here, we found two significant predictors of ENDS ever-use, four significant predictors of current use, and no significant predictors of susceptibility at our adjusted threshold of *p* < 0.005. Higher adulthood SES decreased the likelihood of ENDS ever-use and current use, and current smoking increased the likelihood of ever-use and current use. Two additional factors significantly decreased the likelihood of ENDS current use: being in further tertiary education, and having completed tertiary education. Overall, these findings suggest that smokers with more current socioeconomic disadvantage are more likely to be ever-users of ENDS, and those with more current socioeconomic disadvantage or who have not pursued higher education were more likely to be current users of ENDS. Our analysis of susceptibility using three categorization groups found no significant demographic predictors of susceptibility (Supplementary Table 3).

**TABLE 2:** Results of independent logistic regressions showing estimates in odds ratios (confidence intervals) for demographic predictors of ENDS ever-use, current use, and susceptibility based on the two groups categorization.

Demographic predictors	Ever use [Ref: Never Use] (*n* = 521)	Current use [Ref: Not a current user] (*n* = 521)	Susceptible (two groups) [Ref: Not susceptible] (*n* = 239)
Age	1.049 (0.883–1.246), *p* = 0.587	0.962 (0.770–1.201), *p* = 0.731	0.938 (0.708–1.242), *p* = 0.653
Male–female	0.903 (0.630–1.295), *p* = 0.580	0.909 (0.571–1.449), *p* = 0.689	0.941 (0.510–1.736), *p* = 0.846
Male-gender diverse	3.283 (0.898–12.005), *p* = 0.072	1.090 (0.292–4.065), *p* = 0.898	n/a^3^
Caucasian–Asian	1.022 (0.481–2.169), *p* = 0.956	1.175 (0.460–2.999), *p* = 0.736	1.071 (0.313–3.660), *p* = 0.913
Caucasian-Black	0.510 (0.257–1.010), *p* = 0.054	0.712 (0.267–1.899), *p* = 0.498	0.850 (0.314–2.299), *p* = 0.749
Caucasian-Mixed	1.060 (0.622–1.806), *p* = 0.830	1.504 (0.803–2.819), *p* = 0.203	0.402 (0.132–1.225), *p* = 0.109
Caucasian-Hispanic	0.477 (0.219–1.041), *p* = 0.063	0.348 (0.081–1.504), *p* = 0.158	0.482 (0.133–1.748), *p* = 0.267
Caucasian-Other	1.004 (0.366–2.759), *p* = 0.993	1.567 (0.488–5.025), *p* = 0.450	n/a^3^
SES childhood	1.035 (0.871–1.230), *p* = 0.694	0.862 (0.688–1.081), *p* = 0.199	0.897 (0.668–1.203), *p* = 0.467
SES adulthood	**0.751 (0.630–0.895), *p* = 0.001**	**0.556 (0.433–0.714), *p* < 0.001**	0.886 (0.661–1.188), *p* = 0.419
Employed–Student	0.943 (0.655–1.359), *p* = 0.754	0.805 (0.496-1.307), *p* = 0.381	0.897 (0.489–1.647), *p* = 0.727
Employed–Unemployed	1.657 (0.910–3.017), *p* = 0.099	1.247 (0.620–2.509), *p* = 0.535	0.920 (0.307–2.756), *p* = 0.881
Not in further education-In further education^1^	0.601 (0.377–0.957), *p* = 0.032	**0.428 (0.250–0.732), *p* = 0.002**	0.604 (0.277–1.320), *p* = 0.206
Not in further education-Completed further education^2^	0.568 (0.350–0.922), *p* = 0.022	**0.336 (0.185–0.609), *p* < 0.001**	0.538 (0.237–1.220), *p* = 0.138
Urban–Suburban	1.057 (0.726–1.541), *p* = 0.771	0.822 (0.506–1.336), *p* = 0.429	1.094 (0.578–2.071), *p* = 0.782
Urban–Rural	1.008 (0.602-1.686), *p* = 0.976	0.763 (0.384–1.516), *p* = 0.440	1.102 (0.466–2.610), *p* = 0.824
Not a current smoker-current smoker	**6.839 (3.183–14.692), *p* < 0.001**	**5.424 (3.084–9.538), *p* < 0.001**	3.053 (0.739–12.602), *p* = 0.123

*^1^From the highest attainment variable; compared those Not in further education (*n* = 112, no education after high school) with those currently In further education (*n* = 229; currently undertaking tertiary-level education).*

*^2^From the highest attainment variable; compared those Not in further education (*n* = 112, no education after high school) with those who have Completed further education (*n* = 180, completed further education and employed or unemployed).*

*^3^Insufficient sample size to conduct analyses. **Bolded**, significant at the adjusted *p* < 0.005.*

Table 3 presents the independent logistic regressions for the psychological predictors. Here, we found nine significant predictors of ever-use, four significant predictors of current use, and three significant predictors of susceptibility at our adjusted threshold of *p* < 0.005. Ever-users had a more distressed psychological profile than current users or ENDS susceptible people. Scoring one standard deviation above the mean in Perceived Stress, Anxiety, or Depressive Symptoms increased the likelihood of ENDS ever-use by 39.4, 41.0, and 49.3%, respectively. Similarly, the one personality trait linked closely with mental health problems, Neuroticism, also increased the likelihood of ENDS ever-use by 36.9% through both of its aspects. Fewer of the mental health variables predicted ENDS current use or susceptibility aside from Perceived Stress increasing the likelihood of current use by 41.5%. Conscientiousness was a significant personality predictor of all three outcomes; higher Conscientiousness decreased the likelihood of ever-use by 28.2%, current use by 45.1%, and susceptibility by 41.9% through one or both aspects. Additionally, our analyses of susceptibility using three-group categorization found that higher Conscientiousness decreased the likelihood of moderate susceptibility by 40.8% [OR(CI) = 0.592 (0.412–0.851), *p* = 0.005] (Supplementary Table 4). Contrary to predictions, Curiosity and Exploration did not predict any of the ENDS measures. Only the Openness aspect of Openness/Intellect predicted increased likelihood of ever-use by 31.8%.

**TABLE 3:** Results of independent logistic regressions showing estimates in odds ratios (confidence intervals) for psychological predictors of ENDS ever-use, current use, and susceptibility.

Psychological variables	Ever use [Ref: Never Use] (*n* = 521)	Current use [Ref: Not a Current User] (*n* = 521)	Susceptibility (two groups) [Ref: Not Susceptible] (*n* = 239)
Perceived stress	**1.394 (1.167–1.666), *p* < 0.001**	**1.415 (1.120–1.787), *p* = 0.004**	1.252 (0.940–1.668), *p* = 0.125
Anxiety	**1.410 (1.178–1.687), *p* < 0.001**	1.300 (1.042–1.620), *p* = 0.020	1.417 (1.062–1.890), *p* = 0.018
Depressive symptoms	**1.493 (1.245–1.790), *p* < 0.001**	1.353 (1.085–1.686), *p* = 0.007	1.233 (0.908–1.674), *p* = 0.179
Curiosity & exploration	1.156 (0.972–1.376), *p* = 0.101	1.068 (0.853–1.337), *p* = 0.568	0.762 (0.561–1.034), *p* = 0.081
Neuroticism (N)	**1.369 (1.147–1.635), *p* = 0.001**	1.191 (0.948–1.496), *p* = 0.133	1.308 (0.969–1.765), *p* = 0.079
N–withdrawal	**1.373 (1.151–1.640), *p* < 0.001**	1.290 (1.024–1.626), *p* = 0.031	1.368 (1.009–1.854), *p* = 0.043
N–volatility	**1.295 (1.086–1.544), *p* = 0.004**	1.071 (0.855–1.341), *p* = 0.550	1.197 (0.895–1.602), *p* = 0.225
Agreeableness (A)	1.062 (0.894–1.263), *p* = 0.491	1.188 (0.943–1.496), *p* = 0.144	0.955 (0.721–1.264), *p* = 0.745
A–compassion	1.106 (0.930–1.314), *p* = 0.254	1.147 (0.909–1.447), *p* = 0.247	0.948 (0.722–1.244), p = 0.699
A–politeness	0.991 (0.834–1.178), *p* = 0.920	1.180 (0.938–1.486), *p* = 0.157	0.979 (0.732–1.311), *p* = 0.889
Conscientiousness (C)	**0.718 (0.601–0.858), *p* < 0.001**	**0.549 (0.429–0.703), *p* < 0.001**	**0.581 (0.425–0.794), *p* = 0.001**
C–industriousness	0.783 (0.657–0.933), *p* = 0.006	**0.625 (0.493–0.794), *p* < 0.001**	**0.648 (0.479–0.875), *p* = 0.005**
C–orderliness	**0.723 (0.605–0.865), *p* < 0.001**	**0.593 (0.469–0.751), *p* < 0.001**	**0.616 (0.449–0.844), *p* = 0.003**
Extraversion (E)	0.989 (0.833–1.176), *p* = 0.903	0.889 (0.710–1.113), *p* = 0.306	0.780 (0.586–1.040), *p* = 0.091
E–enthusiasm	0.894 (0.752–1.063), *p* = 0.206	0.807 (0.644–1.012), *p* = 0.063	0.871 (0.654–1.160), *p* = 0.345
E–Assertiveness	1.099 (0.924–1.307), *p* = 0.285	1.010 (0.807–1.264), *p* = 0.934	0.732 (0.545–0.983), *p* = 0.038
Openness/Intellect (O/I)	1.276 (1.070–1.522), *p* = 0.007	1.194 (0.947–1.504), *p* = 0.133	0.984 (0.742–1.305), *p* = 0.910
O/I–openness	**1.318 (1.105–1.572), *p* = 0.002**	1.293 (1.025–1.632), *p* = 0.030	1.144 (0.852–1.537), *p* = 0.372
O/I–intellect	1.150 (0.967–1.368), *p* = 0.114	1.050 (0.837–1.317), *p* = 0.672	0.872 (0.662–1.148), *p* = 0.329

*Bolded, significant at the adjusted p < 0.005.*

Supplementary Figures 1, 2 visualize the dispersion of these demographic and psychological factors between different ENDS users and reinforce findings from the independent logistic regressions. The box plots for the continuous measures (Supplementary Figure 1) show how different ENDS users varied in their SES, mental health variables, Neuroticism, and Conscientiousness, compared to ENDS never users. The bubble plots for the categorical measures (Supplementary Figure 2) show how different ENDS users varied in their highest educational attainment and current smoking, but few other variables.

### Multiple Logistic Regressions

The multiple logistic regressions found very similar results to the independent logistic regressions (Table 4). When predicting ever-use, the final model showed that current smoking and Anxiety increased the likelihood of ever-use by nearly sixfold and 35.7%, respectively, and Conscientiousness (orderliness aspect) decreased the likelihood of ever-use by 25.9%; however, adulthood SES was no longer a significant predictor in the final model, likely due to covariation between adulthood SES and anxiety. For current ENDS use, the final model showed that current smoking increased the likelihood nearly five-fold, whereas SES and Conscientiousness (Orderliness aspect) reduced the likelihood by 34.5 and 37.5% respectively. Only Conscientiousness reduced the likelihood of susceptibility by 38.7%. Finally, in our susceptibility analyses with three group categorization, only higher Conscientiousness decreased the likelihood of moderate susceptibility by 44.4% [OR(CI) = 0.556 (0.374–0.827), *p* = 0.004] (Supplementary Table 5).

**TABLE 4:** Results of the multiple logistic regression showing estimates in odds ratios (confidence intervals) for ENDS ever-use, current use, and susceptibility based on the two groups categorization.

Predictors of ENDS ever-use (*n* = 521)	B	S.E.	Wald	DF	Sig.	Exp(b)	CI
**Block 0 (no predictors added)**							
Constant	0.165	0.088	3.541	1	*p* = 0.060	1.180	
**Block 1 (adding demographic predictors)**							
Constant	–0.002	0.094	0.000	1	*p* = 0.986	0.998	
Adulthood SES	–0.239	0.093	6.626	1	*p* = 0.010	0.788	0.657–0.945
Current smoker	1.848	0.392	22.191	1	***p* < 0.001**	**6.346**	**2.942–13.690**
**Block 2 (adding psychological predictors)**							
Constant	0.021	0.096	0.048	1	*p* = 0.827	1.021	
Adulthood SES	–0.061	0.104	0.348	1	*p* = 0.556	0.941	0.768–1.153
Current smoker	1.750	0.398	19.362	1	***p* < 0.001**	**5.755**	**2.639–12.548**
Anxiety	0.305	0.104	8.680	1	***p* = 0.003**	**1.357**	**1.108–1.662**
Conscientiousness–orderliness aspect	–0.300	0.098	9.429	1	***p* = 0.002**	**0.741**	**0.612–0.897**
O/I–openness aspect	0.252	0.097	6.702	1	*p* = 0.010	1.286	1.063–1.556

**Predictors of ENDS current use (*n* = 521)**	**B**	**S.E.**	**Wald**	**DF**	**Sig.**	**Exp(b)**	**CI**

**Block 0 (no predictors added)**							
Constant	–1.527	0.114	178.031	1	*p* < 0.001	0.217	
**Block 1 (adding demographic predictors)**							
Constant	–1.885	0.142	176.419	1	*p* < 0.001	0.152	
Adulthood SES	–0.515	0.130	15.752	1	***p* < 0.001**	**0.597**	**0.463–0.770**
Current smoker	1.551	0.297	27.326	1	***p* < 0.001**	**4.718**	**2.637–8.442**
**Block 2 (adding psychological predictors)**							
Constant	–1.956	0.149	172.400	1	*p* < 0.001	0.141	
Adulthood SES	–0.422	0.136	9.672	1	***p* = 0.002**	**0.655**	**0.502–0.855**
Current smoker	1.552	0.307	25.533	1	***p* < 0.001**	**4.723**	**2.586–8.625**
Agreeableness–politeness aspect	0.278	0.130	4.611	1	*p* = 0.032	1.321	1.025–1.703
Conscientiousness–orderliness aspect	–0.470	0.131	12.866	1	***p* < 0.001**	**0.625**	**0.484–0.808**

**Predictors of ENDS susceptibility (two groups) (n = 239)**	***B***	***S.E.***	**Wald**	**DF**	**Sig.**	**Exp(b)**	**CI**

**Block 0 (no predictors added)**							
Constant	–1.121	0.151	55.006	1	*p* < 0.001	0.326	
**Block 1 (adding demographic predictors)**							
**No significant results**							
**Block 2 (adding psychological predictors)**							
Constant	–1.083	0.154	49.156	1	*p* < 0.001	0.339	
Conscientiousness	–0.489	0.162	9.140	1	***p* = 0.003**	**0.613**	**0.447–0.842**

*A, agreeable; C, conscientiousness; O/I, openness/intellect. For each model, we entered all demographic predictors in Block 1 and all psychological predictors in Block 2. Significant predictors within each block were selected using a forward likelihood ration method. Bolded, significant at the adjusted p < 0.005 (excluding constants).*

## Discussion

This study explored the demographic and psychological predictors of ENDS use and susceptibility in 521 young-adult MTurk workers in the United States. Overall, we found more predictors of ENDS ever-use and current use than susceptibility. Ever-users and current users were both demographically and psychologically vulnerable. Demographically, ever-users and current users were more likely to be current smokers, of poor socioeconomic means, and current users were also less likely to have pursued any higher education above the high school level. Psychologically, ever-users were more distressed, higher in neuroticism, less conscientious, and higher in openness, whereas current-users were more stressed and less conscientious. Multiple logistic regression showed the importance of current smoking, anxiety, and conscientiousness predicting ENDS ever use, and current smoking, adulthood SES, and conscientiousness predicting ENDS current use. The only predictor of ENDS susceptibility was lower conscientiousness. This paints an interesting picture of the factors that predict ENDS use and susceptibility, as compared to the known predictors of smoking below.

It is interesting that individuals with a higher adulthood SES were *less* likely to have ever used or be current users of ENDS. These findings are in contrast to previous ENDS literature (Adkison et al., 2013; Glover et al., 2018) and suggest the role of SES in ENDS use is similar to that of smoking, where disadvantaged groups are more likely to smoke and face higher exposure to the harms of tobacco (Hiscock et al., 2012). This is possibly due to the lower ongoing costs of ENDS products over time, in comparison to cigarettes (Cheng et al., 2021). One advantage of our study is that we separated childhood and adulthood SES and found that adulthood SES was the more important factor for young adults’ ENDS use. This might be reasonable, given the age of our participants, as young adults are beginning their own lives away from home and developing greater independence. The fact that adulthood SES continued to predict ENDS current use in the multiple logistic regression suggests that this variable is an important factor in young adults’ ENDS use.

The decision to include students and non-students allowed us to test whether ENDS use is higher in college-attending young adults. We found no differences between current students and non-students. Instead, what mattered more was highest educational attainment. In our independent logistic regressions, individuals currently in further education and who had completed further education were significantly *less* likely to be a current ENDS user than people who stopped their education at the high school level. This suggests that highest education level, not whether or not a person is currently attending university, is a factor for ENDS use in young adults. Highest educational attainment is another marker of adulthood SES, which is probably why educational attainment was not selected in the multiple logistic regressions. Moreover, 79% of our sample was educated beyond the high school level. Although this percentage may seem high, other research shows that 87.6% of 18–24 years olds in the US in 2018 had completed high school and almost 60% were engaging in some form of education beyond the high school level (NCES, 2019). It would be important to replicate these patterns in other samples with more people from lower educational backgrounds.

We also replicated prior research showing that current smoking is a significant predictor of ever and current ENDS use. However, current smoking was not a predictor of ENDS susceptibility. This may be because the percentage of smokers in our sample was quite small (*n* = 62, 11.9%), and most of these smokers had already tried ENDS at least once in their life (*n* = 54, 87.1% of current smokers), which excluded them from the susceptibility analysis. Future research is needed to explore the role of current smoking in ENDS susceptibility in populations with higher smoking rates or among younger adolescents with less exposure to ENDS.

Psychological factors related to mental health were important predictors of ENDS ever-use, and to a lesser extent, current use. The link between ENDS ever-use and poorer mental health, particularly anxiety, maps directly onto the smoking literature, which finds higher rates of smoking among highly stressed and anxious young adults (Ng and Jeffery, 2003; Pedersen and Von Soest, 2009). There was some signal that mental health issues increased susceptibility to ENDS, but the confidence intervals were quite wide so we cannot conclude this.

Our exploratory analysis of the personality predictors of ENDS yielded interesting results. The most consistent finding was that ENDS ever-users, current users, and susceptible people shared one personality characteristic: lower conscientiousness. This maps closely to findings from smoking research (Malouff et al., 2006; Hakulinen et al., 2015), and to the wider literature on higher conscientiousness being linked to positive health behaviors (Bogg and Roberts, 2004). Like smoking, neuroticism also predicted likelihood of ENDS ever-use; however, it appeared that anxiety, not neuroticism, was the more important predictor of ENDS ever-use from the multiple logistic regressions. Furthermore, unlike smoking, we found no evidence for higher extraversion among ENDS users. However, ENDS ever-users were higher in the openness aspect, which suggests that young adults with high levels of openness are more likely to have used ENDS at least once in their lives. These findings are reflected in previous smoking literature, showing that higher openness to experience increases the likelihood of smoking (Zvolensky et al., 2015), as well as lower openness to experience being a predictor of quitting smoking (Leung et al., 2013). Further work is necessary to replicate associations between openness and ENDS use and susceptibility.

There were several limitations of our study. First, our study was a cross-sectional correlational design, which cannot establish causality. Second, our sample size was *n* = 521, which halved for the susceptibility analysis (*n* = 239); therefore, future research should recruit a larger cohort of young adults to determine the reliability of the links found here. Larger samples are especially important when using more granular ENDS susceptibility measures that distinguish between highly and moderately susceptible (as with Strong et al., 2015). Third, we did not ask participants if they had never smoked, therefore, we cannot determine the number of young adults in our sample who have used ENDS but never smoked. Moreover, our smoking status and ENDS use measure did not specifically ask about smoking and vaping in the *past* 30-days, as such, we cannot reliably infer from this data that participants responded to these question in relation to the past 30-days. Future research should implement a more rigorous assessment of current smoking and vaping statuses, and categorize dual-use in order to analyze this important group. Fourth, there may be other psychological or personality measures important to ENDS use that we did not measure, such as motivation to quit smoking or sensation-seeking/experience-seeking. The closest traits we measured were openness to experience and curiosity/exploration, but neither were related to ENDS use or susceptibility. Future research should measure sensation-seeking/experience-seeking, to explicate its mixed links to ENDS use (Biener et al., 2015; Sutfin et al., 2015; Case et al., 2017), especially given the greater variety of flavors ENDS offer compared to cigarettes. Fifth, given the relatively novel use of the susceptibility measure in ENDS use, future research should test the predictive validity of this measure in young adults. Sixth, we only surveyed young adults from the United States, making it harder to generalize to young adults from other countries. This is particularly important as attitudes toward ENDS and regulations around the use of ENDS products are more liberal in the United States than many other countries. It is therefore important to explore these factors in other countries. Seventh, we did not ask participants if despite being employed they were receiving a fair wage; this is particularly important as many individuals are working several jobs and it is important that employment factors are considered. Finally, we acknowledge that the use of MTurk for participant recruitment is controversial. We took several steps to ensure the highest quality of data possible, including blocking duplicate IP addresses, paying all participants, describing the study accurately, and placing attention checks throughout the survey. There may also be issues of generalizability between MTurk young adults and the wider young adult population. Survey data from 2014 found that young adult MTurkers are more educated and less likely to smoke than the US population (Walters et al., 2018). Although our 11.9% smoking rate is higher, not lower, than the 8% current smoking rate for US young adults in 2019 (Cornelius et al., 2020), the education level of our sample does appear high. Fortunately, other literature suggests that personality variables of MTurk workers are mostly equivalent to population norms (except for extraversion; Burnham et al., 2018) and that MTurk data are very similar to data collected through more traditional means, i.e., college students (Berinsky et al., 2012). Nevertheless, given the differences found in MTurk samples compared to the general population, we caution against generalizing these findings beyond the present sample and suggest that future research tests these patterns in other samples and the wider young adult population.

## Conclusion

Our study found that young adult ENDS users (past and current) were both demographically and psychologically vulnerable. This vulnerability was indicated by lower current SES, limited education, poorer mental health scores, and more neurotic and less conscientious personality profiles. Several of these predictors of ENDS use are similar to known predictors of cigarette smoking such as SES, mental health issues, neuroticism, and conscientiousness. However, unlike smoking, which is predicted by several personality traits, lower conscientiousness was the single most important personality trait associated with all ENDS variables, and the only predictor of ENDS susceptibility. Interventions targeting ENDS users or the ENDS-curious should focus on young adults who are economically disadvantaged or who present as less conscientious.

## Data Availability Statement

The datasets presented in this study can be found in online repositories. The names of the repository/repositories and accession number(s) can be found below: the dataset analyzed for this study can be found in the Open Science Framework (OSF) Repository [https://osf.io/svdyj//] under the file 2019_MTurkData_predictors of Vaping manuscript.

## Ethics Statement

The studies involving human participants were reviewed and approved by The “Lifestyles of Young Adults” study was approved by the University of Otago Department of Psychology (Category B Ethics #D17/158), with oversight by the University of Otago Ethics Committee. The participants provided their written informed consent to participate in this study.

## Author Contributions

GT conducted the literature review, analyzed the data, and co-wrote the manuscript. TC conceived idea, supervised the data collection, assisted with data analysis, co-wrote the manuscript, and provided supervisory support to GT. Both authors contributed to the article and approved the submitted version.

## Conflict of Interest

The authors declare that the research was conducted in the absence of any commercial or financial relationships that could be construed as a potential conflict of interest.

## Publisher’s Note

All claims expressed in this article are solely those of the authors and do not necessarily represent those of their affiliated organizations, or those of the publisher, the editors and the reviewers. Any product that may be evaluated in this article, or claim that may be made by its manufacturer, is not guaranteed or endorsed by the publisher.
