# Corrigendum: Psychological and Demographic Predictors of Vaping and Vaping Susceptibility in Young Adults

**DOI:** 10.3389/fpsyg.2022.871241

**Published:** 2022-04-22

**Authors:** Grace E. Teah, Tamlin S. Conner

**Affiliations:** Department of Psychology, University of Otago, Dunedin, New Zealand

**Keywords:** e-cigarette, electronic cigarette, vaping, survey, personality, mental health, young adult, socioeconomic status

In the original article, there was an error in Table 3 and Table 4 as published. The statistics for the Openness and Intellect aspects were reversed due to a coding error. The corrected Table 3 and Table 4 appear below. Amendments have also been made to Supplementary Table 1 and Supplementary Table 4.

**Table 3:** Results of independent logistic regressions showing estimates in odds ratios (confidence intervals) for psychological predictors of ENDS ever-use, current use, and susceptibility.

**Psychological**	**Ever use [Ref: Never Use]**	**Current use [Ref: Not a Current User]**	**Susceptibility (two groups)**
**variables**	**(*n* = 521)**	**(*n* = 521)**	**[Ref: Not Susceptible] (*n* = 239)**
Perceived stress	**1.394 (1.167–1.666)**, ***p*** **<** **0.001**	**1.415 (1.120–1.787)**, ***p*** **=** **0.004**	1.252 (0.940–1.668), *p* = 0.125
Anxiety	**1.410 (1.178–1.687)**, ***p*** **<** **0.001**	1.300 (1.042–1.620), *p* = 0.020	1.417 (1.062–1.890), *p* = 0.018
Depressive symptoms	**1.493 (1.245–1.790)**, ***p*** **<** **0.001**	1.353 (1.085–1.686), *p* = 0.007	1.233 (0.908–1.674), *p* = 0.179
Curiosity & exploration	1.156 (0.972–1.376), *p* = 0.101	1.068 (0.853–1.337), *p* = 0.568	0.762 (0.561–1.034), *p* = 0.081
Neuroticism (N)	**1.369 (1.147–1.635)**, ***p*** **=** **0.001**	1.191 (0.948–1.496), *p* = 0.133	1.308 (0.969–1.765), *p* = 0.079
N–withdrawal	**1.373 (1.151–1.640)**, ***p*** **<** **0.001**	1.290 (1.024–1.626), *p* = 0.031	1.368 (1.009–1.854), *p* = 0.043
N–volatility	**1.295 (1.086–1.544)**, ***p*** **=** **0.004**	1.071 (0.855–1.341), *p* = 0.550	1.197 (0.895–1.602), *p* = 0.225
Agreeableness (A)	1.062 (0.894–1.263), *p* = 0.491	1.188 (0.943–1.496), *p* = 0.144	0.955 (0.721–1.264), *p* = 0.745
A–compassion	1.106 (0.930–1.314), *p* = 0.254	1.147 (0.909–1.447), *p* = 0.247	0.948 (0.722–1.244), *p* = 0.699
A–politeness	0.991 (0.834–1.178), *p* = 0.920	1.180 (0.938–1.486), *p* = 0.157	0.979 (0.732–1.311), *p* = 0.889
Conscientiousness (C)	**0.718 (0.601–0.858)**, ***p*** **<** **0.001**	**0.549 (0.429–0.703)**, ***p*** **<** **0.001**	**0.581 (0.425–0.794)**, ***p*** **=** **0.001**
C–industriousness	0.783 (0.657–0.933), *p* = 0.006	**0.625 (0.493–0.794)**, ***p*** **<** **0.001**	**0.648 (0.479–0.875)**, ***p*** **=** **0.005**
C–orderliness	**0.723 (0.605–0.865)**, ***p*** **<** **0.001**	**0.593 (0.469–0.751)**, ***p*** **<** **0.001**	**0.616 (0.449–0.844)**, ***p*** **=** **0.003**
Extraversion (E)	0.989 (0.833–1.176), *p* = 0.903	0.889 (0.710–1.113), *p* = 0.306	0.780 (0.586–1.040), *p* = 0.091
E–enthusiasm	0.894 (0.752–1.063), *p* = 0.206	0.807 (0.644–1.012), *p* = 0.063	0.871 (0.654–1.160), *p* = 0.345
E–Assertiveness	1.099 (0.924–1.307), *p* = 0.285	1.010 (0.807–1.264), *p* = 0.934	0.732 (0.545–0.983), *p* = 0.038
Openness/intellect (O/I)	1.276 (1.070–1.522), *p* = 0.007	1.194 (0.947–1.504), *p* = 0.133	0.984 (0.742–1.305), *p* = 0.910
O/I–openness	**1.318 (1.105–1.572)**, ***p*** **=** **0.002**	1.293 (1.025–1.632), *p* = 0.030	1.144 (0.852–1.537), *p* = 0.372
O/I–intellect	1.150 (0.967–1.368), *p* = 0.114	1.050 (0.837–1.317), *p* = 0.672	0.872 (0.662–1.148), *p* = 0.329

*Bolded, significant at the adjusted p < 0.005*.

**Table 4:** Results of the multiple logistic regression showing estimates in odds ratios (confidence intervals) for ENDS ever-use, current use, and susceptibility based on the two groups categorization.

**Predictors of ENDS ever-use (*n* = 521)**	**B**	**S.E**.	**Wald**	**DF**	**Sig**.	**Exp(b)**	**CI**
**Block 0 (no predictors added)**							
Constant	0.165	0.088	3.541	1	*p* = 0.060	1.180	
**Block 1 (adding demographic predictors)**							
Constant	−0.002	0.094	0.000	1	*p* = 0.986	0.998	
Adulthood SES	−0.239	0.093	6.626	1	*p* = 0.010	0.788	0.657–0.945
Current smoker	1.848	0.392	22.191	1	***p*** **<** **0.001**	**6.346**	**2.942–13.690**
**Block 2 (adding psychological predictors)**							
Constant	0.021	0.096	0.048	1	*p* = 0.827	1.021	
Adulthood SES	−0.061	0.104	0.348	1	*p* = 0.556	0.941	0.768–1.153
Current smoker	1.750	0.398	19.362	1	***p*** **<** **0.001**	**5.755**	**2.639–12.548**
Anxiety	0.305	0.104	8.680	1	***p*** **=** **0.003**	**1.357**	**1.108–1.662**
Conscientiousness–orderliness aspect	−0.300	0.098	9.429	1	***p*** **=** **0.002**	**0.741**	**0.612–0.897**
O/I–openness aspect	0.252	0.097	6.702	1	*p* = 0.010	1.286	1.063–1.556
**Predictors of ENDS current use (*****n*** **=** **521)**	**B**	**S.E**.	**Wald**	**DF**	**Sig**.	**Exp(b)**	**CI**
**Block 0 (no predictors added)**							
Constant	−1.527	0.114	178.031	1	*p* < 0.001	0.217	
**Block 1 (adding demographic predictors)**							
Constant	−1.885	0.142	176.419	1	*p* < 0.001	0.152	
Adulthood SES	−0.515	0.130	15.752	1	***p*** **<** **0.001**	**0.597**	**0.463–0.770**
Current smoker	1.551	0.297	27.326	1	***p*** **<** **0.001**	**4.718**	**2.637–8.442**
**Block 2 (adding psychological predictors)**							
Constant	−1.956	0.149	172.400	1	*p* < 0.001	0.141	
Adulthood SES	−0.422	0.136	9.672	1	***p*** **=** **0.002**	**0.655**	**0.502–0.855**
Current smoker	1.552	0.307	25.533	1	***p*** **<** **0.001**	**4.723**	**2.586–8.625**
Agreeableness–politeness aspect	0.278	0.130	4.611	1	*p* = 0.032	1.321	1.025–1.703
Conscientiousness–orderliness aspect	−0.470	0.131	12.866	1	***p*** **<** **0.001**	**0.625**	**0.484–0.808**
**Predictors of ENDS susceptibility (two groups) (*****n*** **=** **239)**	**B**	**S.E**.	**Wald**	**DF**	**Sig**.	**Exp(b)**	**CI**
**Block 0 (no predictors added)**							
Constant	−1.121	0.151	55.006	1	*p* < 0.001	0.326	
**Block 1 (adding demographic predictors)**							
**No significant results**							
**Block 2 (adding psychological predictors)**							
Constant	−1.083	0.154	49.156	1	*p* < 0.001	0.339	
Conscientiousness	−0.489	0.162	9.140	1	***p*** **=** **0.003**	**0.613**	**0.447–0.842**

*A, agreeable; C, conscientiousness; O/I, openness/intellect*.

*For each model, we entered all demographic predictors in Block 1 and all psychological predictors in Block 2. Significant predictors within each block were selected using a forward likelihood ration method*.

*Bolded, significant at the adjusted p < 0.005 (excluding constants)*.

**Supplementary Table 1:** Descriptive statistics for the psychological variables (*n* = 521).

**Psychological variables**	**N items**	**Response options**	**Possible range**	**Observed range**	**Mean (SD)**	**Cronbach's α**
Perceived stress	10	0 (Never) to 4 (Very often)	0.00–40.00	0.00–40.00	19.72 (8.73)	0.916
Anxiety	7	0 (Not at all) to 3 (Most of the time)	0.00–21.00	0.00–21.00	7.71 (5.04)	0.887
Depressive symptoms	20	0 (Rarely or none of the time (<1 day )) to 3 (Most of or all of the time (5–7 days))	0.00–60.00	0.00–57.00	20.35 (13.62)	0.943
Curiosity and Exploration	10	1 (Very slightly or Not at all) to 5 (Extremely)	1.00–5.00	1.00–5.00	3.07 (0.86)	0.902
Neuroticism (N)	20	1 (Strongly disagree) to 5 (Strongly agree)	1.00–5.00	1.00–5.00	3.04 (0.85)	0.940
N - Withdrawal	10	1 (Strongly disagree) to 5 (Strongly agree)	1.00–5.00	1.00–5.00	3.18 (0.93)	0.902
N - Volatility	10	1 (Strongly disagree) to 5 (Strongly agree)	1.00–5.00	1.00–5.00	2.90 (0.93)	0.920
Agreeableness (A)	20	1 (Strongly disagree) to 5 (Strongly agree)	1.00–5.00	2.00–5.00	3.82 (0.61)	0.889
A - Compassion	10	1 (Strongly disagree) to 5 (Strongly agree)	1.00–5.00	1.00–5.00	3.82 (0.79)	0.908
A - Politeness	10	1 (Strongly disagree) to 5 (Strongly agree)	1.00–5.00	1.80–5.00	3.82 (0.61)	0.766
Conscientiousness (C)	20	1 (Strongly disagree) to 5 (Strongly agree)	1.00–5.00	1.55–4.85	3.31 (0.62)	0.873
C - Industriousness	10	1 (Strongly disagree) to 5 (Strongly agree)	1.00–5.00	1.20–5.00	3.13 (0.82)	0.885
C - Orderliness	10	1 (Strongly disagree) to 5 (Strongly agree)	1.00–5.00	1.80–5.00	3.48 (0.64)	0.722
Extraversion (E)	20	1 (Strongly disagree) to 5 (Strongly agree)	1.00–5.00	1.20–5.00	3.13 (0.68)	0.903
E - Enthusiasm	10	1 (Strongly disagree) to 5 (Strongly agree)	1.00–5.00	1.20–5.00	3.17 (0.79)	0.865
E - Assertiveness	10	1 (Strongly disagree) to 5 (Strongly agree)	1.00–5.00	1.00–5.00	3.08 (0.78)	0.875
Openness/Intellect (O/I)	20	1 (Strongly disagree) to 5 (Strongly agree)	1.00–5.00	1.75–5.00	3.76 (0.57)	0.866
O/I - Openness	10	1 (Strongly disagree) to 5 (Strongly agree)	1.00–5.00	1.60–5.00	3.77 (0.66)	0.855
O/I - Intellect	10	1 (Strongly disagree) to 5 (Strongly agree)	1.00–5.00	1.10–5.00	3.75 (0.70)	0.800

*Measures used: Perceived stress, Perceived stress scale; Anxiety, Hospital anxiety and depression scale (Anxiety sub-scale); Depressive symptoms, Center for epidemiological studies depression scale; Curiosity and exploration, Curiosity and exploration scale-2; Neuroticism to openness/intellect, Big five aspects scale*.

**Supplementary Table 4:** Results of independent logistic regressions showing estimates in odds ratios (Confidence intervals) for psychological predictors of ends susceptibility based on the three groups categorization.

**Psychological predictors**	**Highly susceptible**	**Moderately susceptible**
	**[Ref: Not susceptible] (*n* = 200)**	**[Ref: Not susceptible] (*n* = 217)**
Perceived stress	1.246 (0.814–1.909), p = .311	1.238 (0.886–1.731), p = .211
Anxiety	1.294 (0.830–2.015), p = .255	1.480 (1.060–2.067), p = .021
Depressive symptoms	1.242 (0.786–1.962), p = .353	1.216 (0.852–1.735), p = .282
Curiosity and Exploration	0.616 (0.387–0.981), p = .041	0.869 (0.607–1.244), p = .442
Neuroticism (N)	1.400 (0.888–2.207), p = .147	1.248 (0.879–1.773), p = .216
N - Withdrawal	1.256 (0.800–1.971), p = .321	1.427 (0.993–2.051), p = .055
N - Volatility	1.468 (0.935–2.305), p = .096	1.066 (0.757–1.503), p = .713
Agreeableness (A)	0.610 (0.402–0.924), p = .020	1.269 (0.891–1.808), p = .186
A - Compassion	0.640 (0.434–0.942), p = .024	1.234 (0.869–1.753), p = .241
A - Politeness	0.678 (0.439–1.046), p = .079	1.225 (0.856–1.754), p = .268
Conscientiousness (C)	0.572 (0.356–0.919), p = .021	**0.592 (0.412–0.851), p** **=** **.005**
C - Industriousness	0.656 (0.416–1.034), p = .069	0.647 (0.454–0.922), p = .016
C - Orderliness	0.595 (0.371–0.957), p = .032	0.637 (0.442–0.918), p = .016
Extraversion (E)	0.773 (0.507–1.179), p = .232	0.794 (0.566–1.113), p = .181
E - Enthusiasm	0.814 (0.526–1.259), p = .354	0.909 (0.650–1.271), p = .576
E - Assertiveness	0.767 (0.500–1.175), p = .222	0.724 (0.512–1.025), p = .068
Openness/Intellect (O/I)	0.714 (0.471–1.083), p = .113	1.202 (0.850–1.701), p = .298
O/I - Openness	0.844 (0.542–1.313), p = .452	1.361 (0.952–1.947), p = .091
O/I - Intellect	0.672 (0.447–1.011), p = .056	1.025 (0.735–1.429), p = .855

*Bolded, significant at the adjusted p < .005*.

Due to this coding error, there was also an error in Results, Independent Logistic Regression, Paragraph 2. The Openness and Intellect aspects were reversed. The corrected paragraph is below.

Table 3 presents the independent logistic regressions for the psychological predictors. Here, we found nine significant predictors of ever-use, four significant predictors of current use, and three significant predictors of susceptibility at our adjusted threshold of *p* < 0.005. Ever-users had a more distressed psychological profile than current users or ENDS susceptible people. Scoring one standard deviation above the mean in Perceived Stress, Anxiety, or Depressive Symptoms increased the likelihood of ENDS ever-use by 39.4, 41.0, and 49.3%, respectively. Similarly, the one personality trait linked closely with mental health problems, Neuroticism, also increased the likelihood of ENDS ever-use by 36.9% through both of its aspects. Fewer of the mental health variables predicted ENDS current use or susceptibility aside from Perceived Stress increasing the likelihood of current use by 41.5%. Conscientiousness was a significant personality predictor of all three outcomes; higher Conscientiousness decreased the likelihood of ever-use by 28.2%, current use by 45.1%, and susceptibility by 41.9% through one or both aspects. Additionally, our analyses of susceptibility using three-group categorization found that higher Conscientiousness decreased the likelihood of moderate susceptibility by 40.8% [OR(CI) = 0.592 (0.412–0.851), *p* = 0.005] (Supplementary Table 4). Contrary to predictions, Curiosity and Exploration did not predict any of the ENDS measures. Only the Openness aspect of Openness/Intellect predicted increased likelihood of ever-use by 31.8%.

Due to this coding error, there was also an error in Discussion, Paragraph 1. The Openness and Intellect aspects were reversed. The corrected paragraph is below.

This study explored the demographic and psychological predictors of ENDS use and susceptibility in 521 young-adult MTurk workers in the United States. Overall, we found more predictors of ENDS ever-use and current use than susceptibility. Ever-users and current users were both demographically and psychologically vulnerable. Demographically, ever-users and current users were more likely to be current smokers, of poor socioeconomic means, and current users were also less likely to have pursued any higher education above the high school level. Psychologically, ever-users were more distressed, higher in neuroticism, less conscientious, and higher in openness, whereas current-users were more stressed and less conscientious. Multiple logistic regression showed the importance of current smoking, anxiety, and conscientiousness predicting ENDS ever use, and current smoking, adulthood SES, and conscientiousness predicting ENDS current use. The only predictor of ENDS susceptibility was lower conscientiousness. This paints an interesting picture of the factors that predict ENDS use and susceptibility, as compared to the known predictors of smoking below.

Due to this coding error, there was also an error in Discussion, Paragraph 6. The Openness and Intellect aspects were reversed. In addition Discussion, Paragraph 6 did not address two pieces of literature: Zvolensky et al. (2015) and Leung et al. (2013). The corrected paragraph is below.

Our exploratory analysis of the personality predictors of ENDS yielded interesting results. The most consistent finding was that ENDS ever-users, current users, and susceptible people shared one personality characteristic: lower conscientiousness. This maps closely to findings from smoking research (Malouff et al., 2006; Hakulinen et al., 2015), and to the wider literature on higher conscientiousness being linked to positive health behaviors (Bogg and Roberts, 2004). Like smoking, neuroticism also predicted likelihood of ENDS ever-use; however, it appeared that anxiety, not neuroticism, was the more important predictor of ENDS ever-use from the multiple logistic regressions. Furthermore, unlike smoking, we found no evidence for higher extraversion among ENDS users. However, ENDS ever-users were higher in the openness aspect, which suggests that young adults with high levels of openness are more likely to have used ENDS at least once in their lives. These findings are reflected in previous smoking literature, showing that higher openness to experience increases the likelihood of smoking (Zvolensky et al., 2015), as well as lower openness to experience being a predictor of quitting smoking (Leung et al., 2013). Further work is necessary to replicate associations between openness and ENDS use and susceptibility.

The authors apologize for these errors and state that they do not change the primary scientific conclusions of the article. The original article has been updated.

## Publisher's Note

All claims expressed in this article are solely those of the authors and do not necessarily represent those of their affiliated organizations, or those of the publisher, the editors and the reviewers. Any product that may be evaluated in this article, or claim that may be made by its manufacturer, is not guaranteed or endorsed by the publisher.
