# Supplementary material for: Psychological and Demographic Predictors of Vaping and Vaping Susceptibility in Young Adults
Source: Front Psychol. 2021 Aug 17;12:659206. doi: 10.3389/fpsyg.2021.659206 (PMC8415821; doi:10.3389/fpsyg.2021.659206)
Supplement: Supplementary file 1 [file Data_Sheet_1.pdf]

Supplementary Material

Supplementary Appendix 1

Demographic, Lifestyle, Smoking, and Vaping Survey Questions Used in Analysis

Age

Question: How old are you?

Response Options (single selection):

1. ≤17
2. 18
3. 19
4. 20
5. 21
6. 22
7. 23
8. 24
9. 25
10. 26+

Gender

Question: What is your gender?

Response Options (single selection):

1. Male
2. Female
3. Gender Diverse [Please describe (optional): _______________]

Ethnicity

Question: How would you describe your ethnicity?

Response Options (multiple selection):

1. Aboriginal
2. African
3. Asian
4. Black/African-American/African-British
5. Caucasian/White/European origin
6. Hispanic/Latino
7. Indian
8. Māori
9. Native American

10. Pacific Islander
11. Persian/Arabic
12. Other (Free-text)

Level of Education

Question: Please describe your level of education.

Response Options (single selection):

1. Did not complete high school;
2. Completed high school;
3. Currently attending University, Polytechnic, or other tertiary institution for undergraduate degree;
4. Completed undergraduate degree at University, Polytechnic or other tertiary institution
5. Currently attending University, Polytechnic, or other tertiary institution for higher degree
6. Completed higher degree at University, Polytechnic or other tertiary institution

Student / Employment Status Variable

Question: What is your current employment status?

Response Options (multiple selection):

1. Full-Time Student
2. Part-Time Student
3. Full-Time Work
4. Part-Time Work
5. Unemployed

Notes:

1. For analysis of **Student / Employment status**, we made this into a single variable with the categories of Student, Employed, or Unemployed. For the few cases of multiple endorsements, we prioritized student status such that Student was anyone who reported being a Full-Time Student or Part-Time Student. Employed was anyone not a student who reported being in Full-Time Work or Part-Time Work. Unemployed was anyone who reported being Unemployed without being a student or working.
2. The **Level of Education** and **Student/Employment Status** variables were combined using crosstabs to create a **Highest Attainment** variable analysed in the main manuscript.

Location

Question: Which of the following best describes the area you live in?

1. Urban
2. Suburban
3. Rural
4. Unsure – please describe (Free Text)

Childhood Socioeconomic Status (SES)

Question: We would like to learn more about your CHILDHOOD economic background. If your parents were divorced/separated or permanently stopped living together before you were 18 years old, please answer the following questions for the household in which you spent the most time as a child.

SES 1: My family usually had enough money for things when I was growing up.
SES 2: I grew up in a relatively wealthy neighborhood.
SES 3: I felt relatively wealthy compared to the other kids in my high school.

Response Options (single selection):

1. Strongly Disagree
2. Disagree
3. Slightly Disagree
4. Neither Agree or Disagree
5. Slightly Agree
6. Agree
7. Strongly Agree

Current Socioeconomic Status (SES)

Question: How is your **current** economic situation?

SES 4: I have enough money to buy things I want.
SES 5: I don't need to worry too much about paying my bills.
SES 6: I don't think I'll have to worry about money too much in the future.

Response Options (single selection):

1. Strongly Disagree
2. Disagree
3. Slightly Disagree
4. Neither Agree or Disagree
5. Slightly Agree
6. Agree
7. Strongly Agree

Smoking and Vaping Questions

Question: How often do you currently smoke cigarettes (rolled or filtered)?

Response Options (single selection):

1. I don't smoke now
2. Less than once a month
3. At least once a month
4. At least once a week
5. At least once a day

Have you ever used e-cigarette or vaping device, even one time?

YES (1) NO (0)

How often do you CURRENTLY use e-cigarettes or vaping devices?

1. Not at all
2. Less than monthly
3. At least once a month, but not every week
4. At least once a week, but not every day
5. Every day

If a friend offered you his or her vape, would you puff on it?

1. Definitely Yes
2. Probably Yes
3. Probably No
4. Definitely No

Do you think you are likely to try a vape within the next six months?

1. Definitely Yes
2. Probably Yes
3. Probably No
4. Definitely No

Supplementary Figure Captions

Supplementary Figure 1

Box plots showing dispersion of the continuous measures for different users of electronic nicotine delivery systems (ENDS), current smokers, and all participants. From left to right: dark green = ENDS Never Users (n=239), light green = ENDS Susceptible (n=61), light purple = ENDS Ever Users (n = 282), dark purple = ENDS Current Users (n=93), red = Current Smokers (n=62), grey = all participants (N=521). Each box shows the 25th to 75th percentile; the horizontal line is the median.

Supplementary Figure 2

Bubble plot showing dispersion of the categorical measures for different users of electronic nicotine delivery systems (ENDS), current smokers, and all participants. Columns from left to right: dark green = ENDS Never Users (n=239), light green = ENDS Susceptible (n=61), light purple = ENDS Ever Users (n = 282), dark purple = ENDS Current Users (n=93), red = Current Smokers (n=62), grey = all participants (N=521).

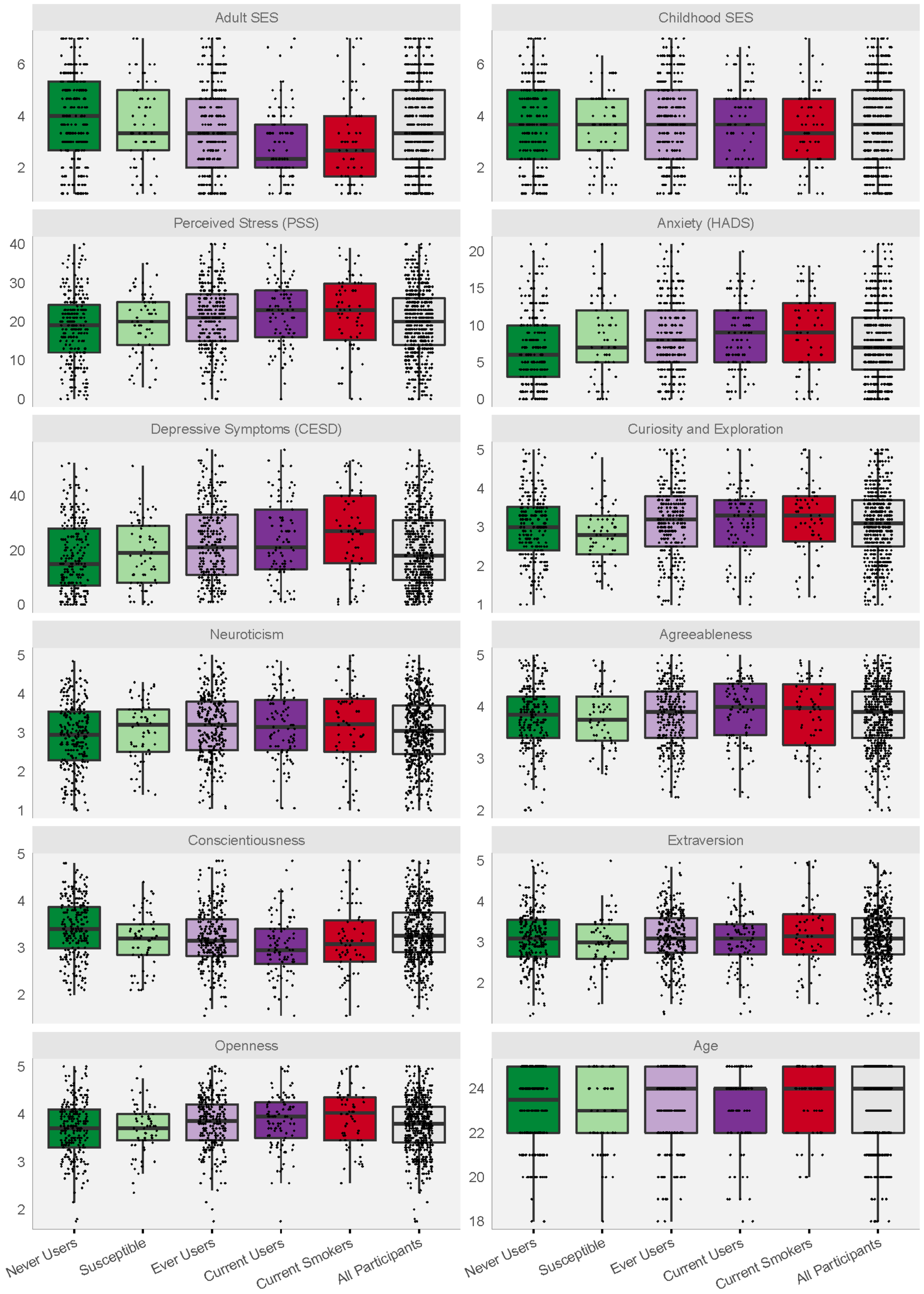
Adult SES
Childhood SES
Perceived Stress (PSS)
Anxiety (HADS)
Depressive Symptoms (CESD)
Curiosity and Exploration
Neuroticism
Agreeableness
Conscientiousness
Extraversion
Openness
Age
Never Users
Susceptible
Ever Users
Current Users
Current Smokers
All Participants

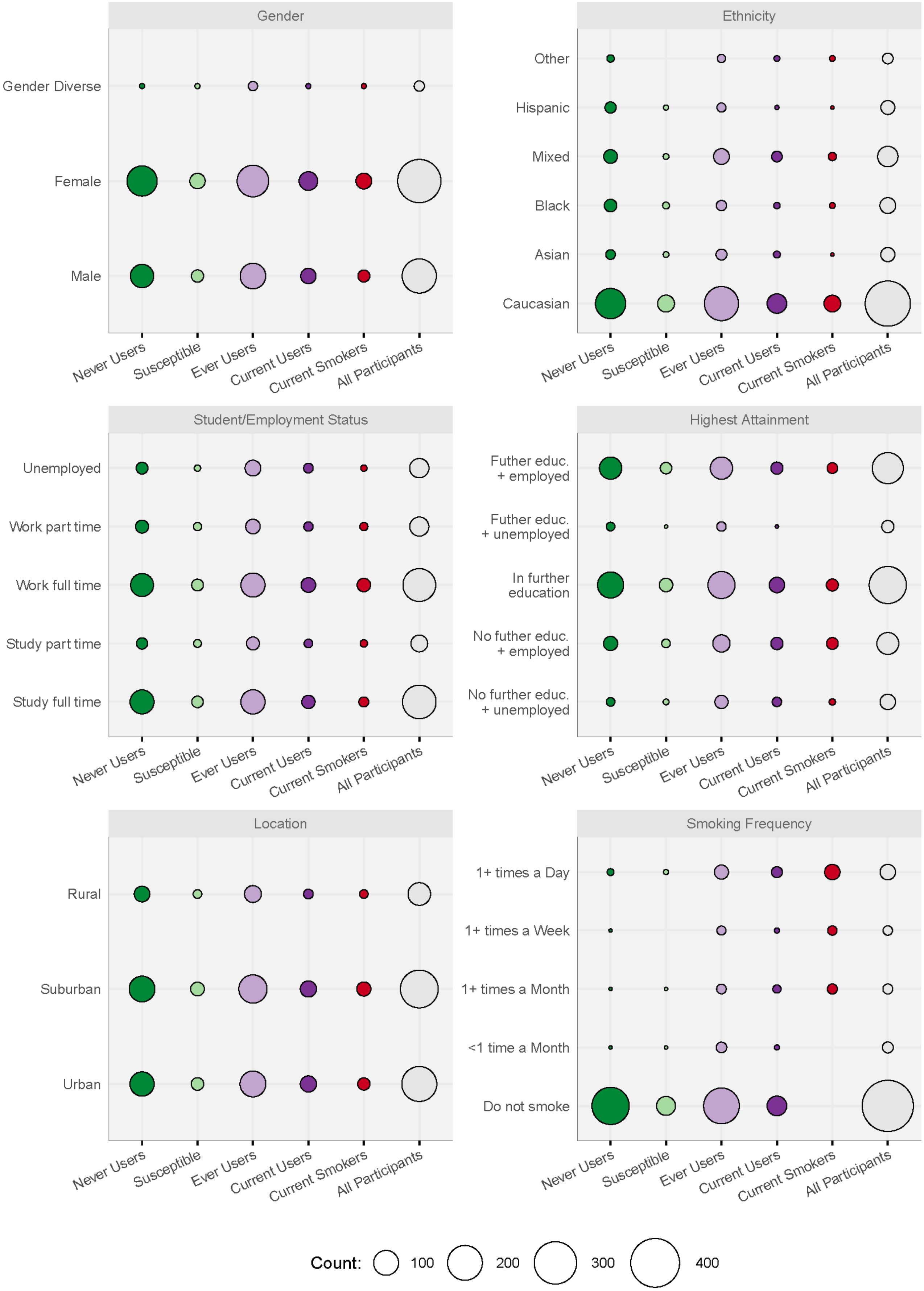
Gender
Gender Diverse
Female
Male
Ethnicity
Other
Hispanic
Mixed
Black
Asian
Caucasian
Student/Employment Status
Unemployed
Work part time
Work full time
Study part time
Study full time
Highest Attainment
Futher educ. + employed
Futher educ. + unemployed
In further education
No futher educ. + employed
No further educ. + unemployed
Location
Rural
Suburban
Urban
Smoking Frequency
1+ times a Day
1+ times a Week
1+ times a Month
<1 time a Month
Do not smoke
Never Users
Susceptible
Ever Users
Current Users
Current Smokers
All Participants
Count: 100 200 300 400

frontiers

Supplementary Table 1

Descriptive Statistics for the Psychological Variables (n=521)

**Psychological Variables**	**N Items**	**Response Options**	**Possible Range**	**Observed Range**	**Mean (SD)**	**Cronbach's α**
Perceived Stress	10	0 (Never) to 4 (Very Often)	0.00-40.00	0.00-40.00	19.72 (8.73)	0.916
Anxiety	7	0 (Not at all) to 3 (Most of the Time)	0.00-21.00	0.00-21.00	7.71 (5.04)	0.887
Depressive Symptoms	20	0 (Rarely or none of the time (< 1 day)) to 3 (Most of or all of the time (5-7 days))	0.00-60.00	0.00-57.00	20.35 (13.62)	0.943
Curiosity & Exploration	10	1 (Very Slightly or Not at All) to 5 (Extremely)	1.00-5.00	1.00-5.00	3.07 (0.86)	0.902
Neuroticism (N)	20	1 (Strongly Disagree) to 5 (Strongly Agree)	1.00-5.00	1.00-5.00	3.04 (0.85)	0.940
N - Withdrawal	10	1 (Strongly Disagree) to 5 (Strongly Agree)	1.00-5.00	1.00-5.00	3.18 (0.93)	0.902
N - Volatility	10	1 (Strongly Disagree) to 5 (Strongly Agree)	1.00-5.00	1.00-5.00	2.90 (0.93)	0.920
Agreeableness (A)	20	1 (Strongly Disagree) to 5 (Strongly Agree)	1.00-5.00	2.00-5.00	3.82 (0.61)	0.889
A - Compassion	10	1 (Strongly Disagree) to 5 (Strongly Agree)	1.00-5.00	1.00-5.00	3.82 (0.79)	0.908
A - Politeness	10	1 (Strongly Disagree) to 5 (Strongly Agree)	1.00-5.00	1.80-5.00	3.82 (0.61)	0.766
Conscientiousness (C)	20	1 (Strongly Disagree) to 5 (Strongly Agree)	1.00-5.00	1.55-4.85	3.31 (0.62)	0.873
C - Industriousness	10	1 (Strongly Disagree) to 5 (Strongly Agree)	1.00-5.00	1.20-5.00	3.13 (0.82)	0.885
C - Orderliness	10	1 (Strongly Disagree) to 5 (Strongly Agree)	1.00-5.00	1.80-5.00	3.48 (0.64)	0.722
Extraversion (E)	20	1 (Strongly Disagree) to 5 (Strongly Agree)	1.00-5.00	1.20-5.00	3.13 (0.68)	0.903
E - Enthusiasm	10	1 (Strongly Disagree) to 5 (Strongly Agree)	1.00-5.00	1.20-5.00	3.17 (0.79)	0.865
E - Assertiveness	10	1 (Strongly Disagree) to 5 (Strongly Agree)	1.00-5.00	1.00-5.00	3.08 (0.78)	0.875
Openness/Intellect (O/I)	20	1 (Strongly Disagree) to 5 (Strongly Agree)	1.00-5.00	1.75-5.00	3.76 (0.57)	0.866
O/I - Openness	10	1 (Strongly Disagree) to 5 (Strongly Agree)	1.00-5.00	1.60-5.00	3.77 (0.66)	0.855
O/I - Intellect	10	1 (Strongly Disagree) to 5 (Strongly Agree)	1.00-5.00	1.10-5.00	3.75 (0.70)	0.800

Measures used: Perceived Stress = Perceived Stress Scale; Anxiety = Hospital Anxiety & Depression Scale (Anxiety Sub-scale); Depressive Symptoms = Center for Epidemiological Studies Depression Scale; Curiosity and Exploration = Curiosity & Exploration Scale-2; Neuroticism to Openness/Intellect = Big Five Aspects Scale.

Supplementary Table 2

Correlations among the continuous predictor variables and current smoking status (0 not smoker, 1 smoker) (n=521)

	Age	SES Adult	SES Child	Current Smoker	Perceived Stress	Anxiety	Depress	Curiosity & Exp	Neur	Agree	Consc	Extra
Age	1.000											
SES Adulthood	.002	1.000										
SES Childhood	-.045	**.315*****	1.000									
Current Smoker	.057	**-.133****	-.058	1.000								
Perceived Stress	-.055	**-.501*****	**-.180*****	.093*	1.000							
Anxiety	-.037	**-.385*****	-.117**	.095*	**.744*****	1.000						
Depressive Symptoms	-.054	**-.498*****	**-.189*****	**.173*****	**.823*****	**.771*****	1.000					
Curiosity & Exploration	.031	**.143****	.111*	.073	**-.305*****	**-.269*****	**-.256*****	1.000				
Neuroticism	-.024	**-.334*****	**-.135****	.048	**.798*****	**.740*****	**.746*****	**-.401*****	1.000			
Agreeableness	.070	-.051	-.072	.021	**-.180*****	-.063	**-.139****	.078	**-.193*****	1.000		
Conscientiousness	.030	**.364*****	.117**	-.090*	**-.532*****	**-.382*****	**-.493*****	**.179*****	**-.514*****	**.244*****	1.000	
Extraversion	.054	**.275*****	**.146****	.034	**-.521*****	**-.431*****	**-.529*****	**.559*****	**-.553*****	**.250*****	**.459*****	1.000
Openness	-.017	-.097*	-.022	.105*	**-.123****	-.057	-.085	**.480*****	**-.195*****	**.433*****	**.193*****	**.432*****

*$p < .05$; **$p < .01$; ***$p < .001$. **Bolded** = significant at the adjusted $p < .005$

Supplementary Table 3

Results of Independent Logistic Regressions showing Estimates in Odds Ratios (Confidence Intervals) for Demographic Predictors of ENDS Susceptibility based on the Three Groups categorization.

Demographic Predictors	Highly Susceptible [Ref: Not Susceptible] (n=200) [1]	Moderately Susceptible [Ref: Not Susceptible] (n=217) [2]
Age	0.785 (0.521-1.182), p = .246	1.046 (0.740-1.479), p = .798
Male-Female	0.431 (0.173-1.079), p = .072	1.553 (0.707-3.412), p = .273
Male-Gender Diverse	n/a[1]	n/a[3]
Caucasian-Asian	0.736 (0.87-6.206), p = .778	1.262 (0.320-4.973), p = .740
Caucasian-Black	1.169 (0.308-4.444), p = .819	0.668 (0.183-2.442), p = .542
Caucasian-Mixed	0.276 (0.035-2.184), p = .223	0.473 (0.133-1.686), p = .248
Caucasian-Hispanic	0.442 (0.055-3.576), p = .444	0.505 (0.109-2.338), p = .505
Caucasian-Other	n/a[1]	n/a[3]
SES Childhood	0.863 (0.557-1.336), p = .508	0.922 (0.651-1.305), p = .645
SES Adulthood	0.911 (0.586-1.414), p = .677	0.876 (0.619-1.238), p = .453
Employed-Student	2.249 (0.824-6.141), p = .114	0.545 (0.258-1.149), p = .111
Employed-Unemployed	1.778 (0.327-9.660), p = .505	0.696 (0.185-2.613), p = .591
Not in further education-In further education	1.096 (0.332-3.623), p = .880	0.407 (0.160-1.037), p = .060
Not in further education-Completed further education	0.377 (0.088-1.618), p = .189	0.603 (0.243-1.497), p = .276
Urban-Suburban	1.460 (0.572-3.728), p = .428	0.899 (0.410-1.969), p = .790
Urban-Rural	0.317 (0.038-2.652), p = .289	1.521 (0.597-3.875), p = .379
Not a current smoker-current smoker	4.350 (0.749-25.267), p = .101	2.351 (0.415-13.317), p = .334

[1] Tested 22 Highly Susceptible versus 178 Committed Never ENDS users (total n = 200). [2] Tested 39 Moderately Susceptible versus 178 Committed Never ENDS users (total n = 217). [3] Insufficient sample size to conduct analyses. **Bolded** = significant at the adjusted $p < .005$

Supplementary Table 4

Results of Independent Logistic Regressions showing Estimates in Odds Ratios (Confidence Intervals) for Psychological Predictors of ENDS Susceptibility based on the Three Groups categorization.

**Psychological Predictors**	**Highly Susceptible [Ref: Not Susceptible] (n=200)**	**Moderately Susceptible [Ref: Not Susceptible] (n=217)**
Perceived Stress	1.246 (0.814-1.909), p = .311	1.238 (0.886-1.731), p = .211
Anxiety	1.294 (0.830-2.015), p = .255	1.480 (1.060-2.067), p = .021
Depressive Symptoms	1.242 (0.786-1.962), p = .353	1.216 (0.852-1.735), p = .282
Curiosity & Exploration	0.616 (0.387-0.981), p = .041	0.869 (0.607-1.244), p = .442
Neuroticism (N)	1.400 (0.888-2.207), p = .147	1.248 (0.879-1.773), p = .216
N - Withdrawal	1.256 (0.800-1.971), p = .321	1.427 (0.993-2.051), p = .055
N - Volatility	1.468 (0.935-2.305), p = .096	1.066 (0.757-1.503), p = .713
Agreeableness (A)	0.610 (0.402-0.924), p = .020	1.269 (0.891-1.808), p = .186
A - Compassion	0.640 (0.434-0.942), p = .024	1.234 (0.869-1.753), p = .241
A - Politeness	0.678 (0.439-1.046), p = .079	1.225 (0.856-1.754), p = .268
Conscientiousness (C)	0.572 (0.356-0.919), p = .021	**0.592 (0.412-0.851), p = .005**
C - Industriousness	0.656 (0.416-1.034), p = .069	0.647 (0.454-0.922), p = .016
C - Orderliness	0.595 (0.371-0.957), p = .032	0.637 (0.442-0.918), p = .016
Extraversion (E)	0.773 (0.507-1.179), p = .232	0.794 (0.566-1.113), p = .181
E - Enthusiasm	0.814 (0.526-1.259), p = .354	0.909 (0.650-1.271), p = .576
E - Assertiveness	0.767 (0.500-1.175), p = .222	0.724 (0.512-1.025), p = .068
Openness/Intellect (O/I)	0.714 (0.471-1.083), p = .113	1.202 (0.850-1.701), p = .298
O/I - Openness	0.844 (0.542-1.313), p = .452	1.361 (0.952-1.947), p = .091
O/I - Intellect	0.672 (0.447-1.011), p = .056	1.025 (0.735-1.429), p = .855

Bolded = significant at the adjusted $p < .005$

Supplementary Table 5

Results of the Multiple Logistic Regression Showing Estimates in Odds Ratios (Confidence Intervals) for ENDS Susceptibility based on the Three Groups categorization.

**Predictors of ENDS Highly Susceptible (n=200)**	**B**	**S.E.**	**Wald**	**DF**	**Sig.**	**Exp(b)**	**CI**
Block 0 (No Predictors Added)							
Constant	-2.137	0.231	85.803	1	p < .001	0.118	
Block 1 (Adding Demographic Predictors)							
No significant results							
Block 2 (Adding Psychological Predictors)							
Constant	-2.271	0.255	79.250	1	p < .001	0.103	
Agreeableness - Compassion	-0.441	0.201	4.840	1	p = .028	0.643	0.434-0.953
**Predictors of ENDS Moderately Susceptible (n=217)**	**B**	**S.E.**	**Wald**	**DF**	**Sig.**	**Exp(b)**	**CI**
Block 0 (No Predictors Added)							
Constant	-1.571	0.181	75.589	1	p < .001	0.208	
Block 1 (Adding Demographic Predictors)							
No significant results							
Block 2 (Adding Psychological Predictors)							
Constant	-1.540	0.187	67.764	1	p < .001	0.214	
Agreeableness	0.383	0.196	3.797	1	p = .051	1.466	0.998-2.154
Conscientiousness	-0.586	0.202	8.388	1	**p = .004**	**0.556**	**0.374-0.827**

Bolded = significant at the adjusted $p < .005$ (excluding constants).
